# Cardiopulmonary Test in Fontan Patients: Is the Type of Ergometer Critical?

**DOI:** 10.3390/jcdd12100381

**Published:** 2025-09-25

**Authors:** Federica Gentili, Giulia Cafiero, Eliana Tranchita, Jacopo Kowalczyk, Fausto Badolato, Paola Pagliari, Benedetta Leonardi, Giulio Calcagni, Gabriele Rinelli, Claudia Montanaro, Fabrizio Drago, Ugo Giordano

**Affiliations:** 1 Cardiology and Arrhythmology Complex Unit, Clinical Area of Fetal, Neonatal and Cardiological Sciences, Bambino Gesù Children’s Hospital, IRCCS, 00165 Rome, Italy; 2 Adult Congenital Heart Disease Unit, Clinical Area of Fetal, Neonatal and Cardiological Sciences, Bambino Gesù Children’s Hospital, IRCCS, 00165 Rome, Italy

**Keywords:** cardiopulmonary exercise stress test (CPET), Fontan, congenital heart disease (CHD), cycle ergometer, treadmill

## Abstract

Cardiopulmonary exercise testing (CPET) is recommended as part of routine care in people with congenital heart disease. A significant difference has been observed in many CPET parameters, depending on the ergometer and exercise protocol used. The aim of this study is to investigate such differences in Fontan patients. All Fontan patients (<40 years old, NYHA class I/I–II) underwent two consecutive CPETs on different ergometers (treadmill with ramped Bruce protocol versus cycle ergometer with ramp protocol) within less than 12 months. The exclusion criterion was the presence of significant clinical/anthropometric changes between the two tests. Anthropometric, surgical, clinical, electrocardiogram (ECG) and CPET data were collected. 47 subjects were enrolled (25 males, mean age 16.4 at first test). Peak heart rate (HR) tended to be higher on the treadmill (*p* = 0.05 as % of predicted, *p* = 0.062 in absolute value). Peak oxygen consumption (VO_2_) (mL/min, mL/kg/min, and % of predicted) was significantly higher on the treadmill (*p* < 0.01), as well the VO_2_ at the ventilatory anaerobic threshold (VAT) and the peak oxygen pulse. A different kinetics of the oxygen pulse wave was observed in the same patient comparing the two testing modalities. Maximal respiratory-exchange-ratio values (>1.1) were reached more frequently on the cycle ergometer (*p* < 0.001). The minute ventilation–carbon dioxide output slope (VE/VCO_2_ slope) was not different between the two tests (*p* = 0.400). Many parameters of CPET may differ depending on the ergometer used. These should be considered in clinical evaluation of Fontan patients and when exercise is to be prescribed.

## 1. Introduction

Nowadays, patients with Fontan circulation physiology commonly survive into adulthood, generating new challenges for clinicians. Over time, many comorbidities can be acquired, both within and outside the cardiovascular system [1,2] resulting in deterioration in quality of life, increased unplanned hospitalizations, and sometimes major adverse events such as premature death [3]. For this reason, it is important to identify prognostic factors in this population, i.e., clinical parameters associated with future health status, which can be used for more appropriate risk stratification, to guide treatment choices and to monitor treatment outcome [4,5].

Cardiopulmonary exercise testing (CPET) has emerged as a useful tool in this context: the combination of standard exercise testing (ECG monitoring, O_2_ saturation, chronotropic and pressor response to exercise, and presence of exercise-induced symptoms) and the measurement of ventilatory gas exchange allows us to better identify the pathophysiological mechanism(s) limiting exercise [6,7] and accurately quantify the cardiorespiratory fitness (CRF), i.e., the individual’s ability to perform exercise [8,9], which is now recognized as an important prognostic marker in many cardiorespiratory diseases [3,10,11,12,13,14,15,16]. Growing literature is documenting the potential prognostic role in Fontan patients of many CPET derived parameters, such as peak oxygen consumption (peak VO_2_) [17,18,19], VO_2_ trend over time [20,21], oxygen uptake efficiency slope (OUES) [22,23], VO_2_ at the ventilatory anaerobic threshold (VO_2_ at VAT) [17,18], minute ventilation/carbon dioxide production slope (VE/VCO_2_ slope) [17,22], oxygen pulse pattern [17], and exercise oscillatory ventilation (EOV) [16,24,25].

In addition to its potential prognostic role in Fontan patients, CPET provides valuable information for setting up a physical training program “tailored” to specific disease conditions. Children and adolescents with congenital heart disease (CHD) should be encouraged to adopt a physically active lifestyle [6,26], and this is especially true in individuals with univentricular hearts after Fontan surgery. Exercise seems to be one of the best therapies in this context, being able to act and improve most of the factors that are related to exercise intolerance in these patients [27,28,29], for whom a tailored exercise prescription is strongly recommended [30,31]. Exercise prescription to individuals with heart disease is a rather complicated process [26,32,33], accurate identification of exercise intensity is sometimes complex, and direct detection of the first (VAT) and second (respiratory compensation point) ventilatory thresholds during incremental testing is extremely helpful in this regard [34,35,36].

For all these reasons, CPET is recommended as a part of routine care in people with CHD by the European Society of Cardiology [6,37]. However, there are no indications regarding the type of test to be carried out in Fontan population.

The most commonly available ergometers in cardiology centers are the treadmill and the cycle ergometer, each with its own specific advantages and limitations.

The cycle ergometer usually allows for improved electrocardiographic analysis due to fewer motion-related artifacts from the upper body [38]. It also supports the use of customized ramp protocols, enabling consistent work rate increases at intervals of less than 60 s, and facilitates the clinician’s identification of important parameters, such as the ventilatory anaerobic threshold [39]. Additionally, the incidence of serious adverse events or events requiring medical intervention during exercise testing appears to be lower with the cycle ergometer [40,41].

Treadmill walking and running, on the other hand, is generally considered a more natural form of exercise, particularly suitable for children [42]. It tends to produce higher heart rates and—by engaging more muscle groups—typically results in a higher peak VO_2_, with values reported to be up to 20% higher than those achieved with cycling in children [42,43] and untrained adult individuals [33,44,45,46,47], as in athletes from various sports disciplines [48,49,50].

Of particular relevance to this context, our recent study involving patients with repaired Tetralogy of Fallot (rTOF) demonstrated significant differences in key CPET-derived parameters depending on the type of ergometer and exercise protocol used [51]. To our knowledge, no other studies have compared treadmill versus cycle ergometer CPET in specific populations of patients with congenital heart disease (CHD). However, similar findings have been reported in patients with heart failure and/or ischemic heart disease [52,53,54,55,56]. In the Huertas study conducted on subjects with Heart Failure with Reduced Ejection Fractio (HFrEF), differences in key CPET parameters (including peak VO_2_ and VE/VCO_2_) derived from treadmill and cycle ergometers in the same patient led to a change in functional class of 23 (51%) patients and ventilatory class of 28 (47%) patients, with an important impact on prognostic scales and possible advanced treatment [53].

Therefore, the purpose of the study is to evaluate whether the use of a different ergometer may affect the outcome of the examination in Fontan patients. In our view, this question is particularly important given the established role of CPET in the clinical and prognostic evaluation of these patients

## 2. Materials and Methods

All Fontan patients followed at the cardiology department of the Bambino Gesù Children’s Hospital were evaluated for enrolment in the study.

Inclusion criteria were as follows: age between 8 and 40 years old, stable clinical condition in absence of contraindications to maximal exercise testing, and informed consent of candidates to undergo the study. The exclusion criteria were as follows: a reduced compliance when pedaling a bicycle and/or walking/running on treadmill, a NYHA functional class of I or I–II, the presence of significant changes between the two tests in clinical condition, pharmacological treatment, and anthropometric parameters or lifestyle (different International Physical Activity Questionnaire, IPAQ stage).

All eligible subjects were asked to undergo two consecutive CPETs on different ergometers (treadmill with ramped Bruce protocol versus a cycle ergometer with a ramp protocol) within less than 12 months. The subjects were randomly assigned to make the first CPET on the cycle ergometer or on the treadmill, so that half of the tested patients made the first test cycling. The study was approved by the Ethics Committee of the Bambino Gesù Children’s Hospital, Research Institute (protocol 2083_OPBG_2020), and all subjects signed an informed consent form. The study was conducted in accordance with the Declaration of Helsinki.

First level evaluation: all subjects underwent a complete clinical examination, including a 12-lead electrocardiogram (ECG) and an echocardiography before each CPET. IPAQ was also administered to evaluate the amount of physical activity practiced in the previous week.

Second level evaluation: CPET. All recruited patients were tested over the course of one year with two consecutive CPETs including lung function test using 2 different ergometers (cycle ergometer or treadmill). Before each test, every subject was informed on the characteristics of the test; a snack 2 h before the exam and proper hydration were recommended, such as the regular intake of drug therapy at least 2 h before the examination. The CPET was conducted with a Quark Pulmonary Function Testing (PFT) Metabolic Cart (COSMED, Rome, Italy)**,** regularly calibrated before each test, with breath-by-breath data acquisition, using face masks of suitable size, with continuous 12-lead ECG monitoring, finger oximetry and manual blood pressure (BP) measurement every 2–3 min using a cuff appropriate for the patient’s anthropometric measurements. During the tests, patients exercised until volitional/muscular fatigue or until the possible occurrence of symptoms and/or appearance of threatening arrhythmias (supraventricular or ventricular tachycardia, atrial fibrillation). Tests were considered maximal when at least two of the following criteria were achieved: failure to maintain the work rate; respiratory exchange ratio (RER) > 1.05, or maximal heart rate (HR) >85% of the age-predicted maximum (220 − age) with dyspnea or occurrence of a VO_2_ plateau (VO_2_ increase less than 150 mL/min over the last 30 s of the test). Before each test, a spirometry was performed with the detection of Forced Vital Capacity (FVC), Forced Expiratory Volume in 1 Second (FEV1), and FEV1/FVC ratio.

The following CPET variables were measured: peak oxygen uptake (pVO_2_) in absolute value (mL/min), pro-kilo (mL/kg/min), and % of predicted value (pVO_2_ was calculated in both tests as the 15 s average of the highest VO_2_ achieved during the test); peak oxygen pulse and oxygen pulse kinetics; the slope of the minute ventilation (VE) to CO_2_ production ratio (VE/VCO_2_ slope), evaluated up to the ventilatory compensation point (VCP); peak minute ventilation (peak VE); and HR and oxygen uptake at the ventilatory anaerobic threshold (VAT). VAT was estimated by V slope method and ventilator equivalent method [57]. VCP was identified where VE started to change out of proportion to VCO_2_ production with the increase in VE/VCO_2_ and the consequent decline of the end-tidal partial pressure of carbon dioxide (PetCO_2_) [58,59].

All CPET results were compared to Burstein’s pediatric reference values (<19 years) [60] or Wasserman’s reference values (>19 years) [61] when appropriate.

The analysis was carried out by the same operator for all the tests analyzed.

Statistical Analysis: As most of the continuous data showed a non-Gaussian distribution, data are reported as median value and interquartile range (IQR). Categorical variables are reported as counts and percentages. Normality was assessed using Shapiro–Wilk normality test. To compare CPET on the cycle ergometer with CPET on the treadmill, the Wilcoxon signed-rank test for paired data was used. Chi-square test was used for comparing categorical variables. Statistical significance was considered for a *p* value set at ≤0.05. All statistical analyses were performed using Jamovi Software, version 1.6.23.0 for macOS.

## 3. Results

47 subjects were enrolled in the study (25 males and 22 females, median age at the first test was 16.4), 17 with right a univentricular heart, 24 with a left univentricular heart and 6 with an isomeric ventricle. The main anthropometric characteristics of the test population at the first and second test are shown in Table 1. As required by the inclusion criteria, no subject showed significant changes between the two tests in the clinical picture, therapy, anthropometric parameters, or lifestyle (IPAQ stage), with 19 subjects (40%) resulting inactive (IPAQ < 700) and 28 subjects (60%) sufficiently active (IPAQ 700–2519) both at the first and second test.

Table 2 reports the main CPET parameters on treadmill and cycle ergometer, respectively.

No significant differences in oxygen saturation were observed (*p* > 0.05).

Peak heart rate tended to be higher on the treadmill, with a *p*-value of 0.05 when analyzed as a percentage of the predicted maximum heart rate and a *p*-value of 0.062 when analyzed in absolute terms (bpm).

Peak VO_2_ (mL/min, mL/kg/min, and % of predicted) was significantly higher on the treadmill than on the cycle ergometer (*p* < 0.001); conversely, peak RER values (>1.1) were reached more frequently on the cycle ergometer (*p* < 0.001).

The VO_2_ at VAT (either expressed as mL/min, mL/kg/min, or as a % of the predicted VO_2_ max) was also higher on the treadmill than on the cycle ergometer (*p* < 0.001).

The peak oxygen pulse was statistically higher on the treadmill both as mL/beat (*p* < 0.001) and as percentage of the predicted value (*p* < 0.001). Characteristically, using a different ergometer, a different kinetics of the oxygen pulse wave was observed in the same patient, with a statistically significant prevalence (*p* < 0.001) on the treadmill of an altered pattern (early and sustained plateau/decline) compared to a normal pattern (continuous growth); specifically, 14 subjects showed normal kinetics with both ergometers, 3 had abnormal kinetics regardless of modality, and 30 exhibited abnormal kinetics only on the treadmill.

The VE/VCO_2_ slope did not document significant differences between the two tests (*p* = 0.400).

## 4. Discussion

To the best of our knowledge, this is the first study to assess intra-patient differences in CPET parameters between cycle ergometer and treadmill testing in a population of pediatric and young adult Fontan patients.

The data obtained from both ergometers confirm a reduction in the functional capacity of these patients, consistent with what has already been reported in the literature on patients with CHD and Fontan patients [16,62,63,64,65,66,67]. Both modalities were found to be safe for evaluating functional capacity in this group, with no major adverse events reported with either method.

At the same time, however, our findings documented the presence of statistically significant differences in several CPET-derived parameters in Fontan patients, depending on the type of ergometer used. These findings are in line with those reported in other papers on patients with HF and/or ischemic heart disease [52,53,54,55,56], and in our recent study on rTOF [51]. These differences could carry important prognostic and therapeutic implications, particularly in relation to exercise prescription.

### 4.1. Peak Heart Rate

The peak HR achieved in our cohort was higher on the treadmill, both in absolute terms (*p =* 0.062) and as a percentage of the predicted value (*p =* 0.050). Beyond the statistical data, which are only partially significant, the clinical relevance of these findings appears rather limited, as in 75% of cases the variation was less than 4 bpm. Nevertheless, given the importance of heart rate augmentation during exercise in the clinical and prognostic assessment of Fontan patients, we believe that this difference between the two ergometers, with a tendency toward higher peak HR on the treadmill, deserves attention. In this regard, it should be emphasized that our result is consistent with previous evidence in the literature. Treadmill exercise testing imposes greater cardiovascular stress by engaging a larger number of muscle groups in more natural, weight-bearing, gravity-resisting activities [52]. As a consequence, subjects more frequently achieve higher peak HR values on the treadmill, which is essential for accurately assessing cardiovascular capacity and potential limitations [53]. By contrast, cycle ergometer testing typically elicits lower peak HRs, particularly in populations with specific health conditions or physical limitations. For example, patients with heart failure often exhibit lower peak HRs on the cycle ergometer, which may complicate the interpretation of exercise capacity and the evaluation of the need for further interventions [53]. Peak HR during exercise testing represents a critical parameter for assessing cardiovascular fitness and overall health, and this discrepancy highlights the importance of considering the exercise modality when evaluating peak HR and overall cardiovascular performance.

### 4.2. Peak VO_2_

In our study, peak VO_2_ (mL/min, mL/kg/min, and % of predicted) was significantly higher on the treadmill than on the cycle ergometer (*p* < 0.001). Although the median values observed (VO_2_: 23.2 to 25.6 mL·kg^−1^·min^−1^) does not appear to be clinically meaningful, the finding is consistent with the near-unanimous literature [42,43,44,45,46,47,48,49,50,51,52,53,54,55,56], and is particularly relevant given the important prognostic value of this parameter in this patient subpopulation

In the study by Mazaheri et al. [52] on adult patients with heart failure and severely reduced ejection fraction, peak VO_2_ was 23.12% higher on the treadmill than on the cycle (20.55 ± 3.3 vs. 16.69 ± 3.01, *p* < 0.001, respectively), a percentage similar to that observed by Herrero Huertas et al. [53] in subjects with HFrHF, with peak VO_2_ values 20% higher (*p* < 0.000) on the treadmill, as well as in a second study by the same group [55] in patients with HFrEF and chronic obstructive pulmonary disease (20 ± 5 vs. 17 ± 4 mL/kg/min, *p* < 0.001). Our recent study on subjects with TOF [51] also documented in this population a significantly higher peak oxygen consumption on the treadmill (31.7 ± 6.9 vs. 25.5 ± 5.5; *p* < 0.0001).

Moreover, similar findings had already been reported in untrained healthy individuals [33,44,45,46,47], with differences ranging from 5% to 20%, as well as in the pediatric population [42,43]. As early as 1977, Boileau et al. documented an 8% higher peak VO_2_ in 11- to 14-year-old children during treadmill testing compared with cycle ergometry [43], and more recently (2001), LeMura et al. reported significantly higher HR and peak VO_2_ values on the treadmill in 5- to 6-year-old children [42].

In this regard, it has been emphasized that the choice of reference values can be critical—particularly in children and adolescents with congenital heart disease. To avoid significant errors, the reference values used must closely reflect not only the population under study but also the type of test with which the individual is assessed [68,69].

The only conflicting data comes from studies on professional athletes who specialize in cycling, who exhibit similar peak VO_2_ values on both types of ergometers [48,49,70]. VO_2_ max is a function of the O_2_ transport system, including pulmonary ventilation, cardiac output, and O_2_ extraction from peripheral muscles. Regular training of specific muscle groups with specific athletic gestures (as in the case of cycling or swimming) can influence these processes, for example, by optimizing the response to certain types of exercise by certain types of muscles.

Conversely, the opposite may be true for deconditioned individuals—and even more for patients with CHD (e.g., repaired tetralogy of Fallot or single-ventricle/Fontan physiology)—who are generally sedentary and more accustomed to walking than to cycling.

An additional isolated, partially conflicting finding is derived from a 2004 study of severely overweight youth (12.5 ± 2.8 years) that reported no statistically significant difference in peak VO_2_ between the two testing modalities, although point estimates were higher on the treadmill [71].

### 4.3. Respiratory Exchange Ratio (RER)

The RER is a parameter measured during CPET, reflecting the balance between CO_2_ production and O_2_ consumption.

In the present study, we found more frequently maximal RER values (>1.1) on the cycle ergometer. This result is in line with what has been reported in other studies, which had shown lower peak RER values during treadmill testing than during the cycle ergometer [52,72]. Mazaheri et al. found that patients with heart failure exhibited lower peak RER levels during treadmill tests than during cycle ergometry, suggesting inherent physiological differences in muscle fiber recruitment and energy metabolism between these modalities [52]. In their recent study, Roxburgh et al. have shown that treadmill testing elicited the highest peak VO_2_ but resulted in a lower RER, suggesting that this measure should not be misinterpreted as a lack of exertion [72]. Similar results have been observed in pediatric healthy subjects [42]. This discrepancy is likely due to the different muscle recruitment and the earlier fatigue of the specific muscle groups engaged in the effort, usually not trained in the specific gesture of cycling. Although this divergence in peak RER values appears evident in our routine clinical practice with pediatric patients with a univentricular heart, no significant differences in peak RER values have been observed in some other studies involving patients with heart failure [53,54,55]. Further studies will be useful to clarify this issue.

### 4.4. Ventilatory Anaerobic Threshold (VAT)

The ventilatory anaerobic threshold (VAT) is defined as the exercise intensity at which the body transitions from predominantly aerobic metabolism to anaerobic metabolism, leading to an increase in CO_2_ production relative to O_2_ consumption [63]. VAT is a critical marker of exercise capacity and previous studies proved that VAT is significantly lower in Fontan patients than in healthy controls [73,74,75]. Lower VAT values have been linked to increased morbidity and mortality in this population, emphasizing the importance of regular monitoring and assessment of exercise capacity [74,75]. In addition to its prognostic values, the identification of VAT is extremely helpful for a tailored exercise prescription in subjects with CHD [34].

In our study, an earlier achievement of VAT during tests on the cycle ergometer compared to the treadmill has been observed, with values of VO_2_ (mL/min, mL/kg/min, and % of predicted VO_2_ max) and HR (bpm and % of maximum predicted) significantly lower on the cycle ergometer. Once again, this data appears to be in line with what was observed in the studies already cited on subjects with heart failure [52,53,55], as well as with what we observed in subjects with rTOF [51].

This difference could be explained by a decreased tendency pertaining to the specific type of exertion of this particular population, often characterized by reduced muscle tone, especially of the lower limbs, and certainly more easily accustomed to walking than to cycling at an imposed pace, resulting in a more wasteful technical gesture and consequent earlier fatigue. In support of this explanation, there are some studies in the sports medicine field [48,49,65]. In cyclists rather than in high-level swimmers, for example, the muscles adapt to the specific type of training, resulting in an improvement (for that given exercise) of submaximal physiological variables such as the ventilatory threshold, in some cases without a variation in VO_2_ max. At the same time, training allows you to optimize the recruitment of motor units, making muscle contraction more efficient and the technical gesture less expensive, once again improving submaximal variables such as VAT.

In our view, this finding is of particular importance in the context of exercise prescription and highlights the importance of considering the biomechanical and muscular demands of each ergometer when interpreting VAT result.

### 4.5. VE/VCO_2_ Slope

The VE/VO_2_ slope is a critical parameter that reflects ventilatory efficiency and is associated with exercise capacity and prognosis in cardiovascular diseases and in Fontan patients [17,22,76,77,78].

The difference in the VE/VO_2_ slope during cardiopulmonary exercise testing using various ergometers, such as treadmills and cycle ergometers, has been a subject of investigation in clinical settings, particularly concerning patients with heart failure.

Huertas et al. suggested that exercise modality may influence the VE/VCO_2_ slope in patients with chronic heart failure [53], although subsequent studies on similar patients did not confirm this [52,55]. Likewise, our recent work on patients with repaired tetralogy of Fallot found no significant differences between treadmill and cycle ergometer testing [51]. In our cohort of Fontan patients, the VE/VCO_2_ slope resulted in not being different between the two testing modalities, suggesting that both ergometers can be utilized interchangeably for assessing ventilatory efficiency in these patients.

### 4.6. Peak Oxygen Pulse

The O_2_ pulse, defined as the ratio of O_2_ uptake to HR, is a particularly significant parameter, as it reflects the stroke volume during peak exercise, which is often compromised in subjects with univentricular physiology.

Longitudinal studies have shown that peak O_2_ pulse is usually reduced in Fontan patients and tends to decrease over time, reflecting a deterioration in exercise capacity and cardiovascular function [67].

In our study, the peak O_2_ pulse was significantly higher on treadmill than the cycle ergometer both as a mL/beat (*p* < 0.001) and as a % of the predicted value (*p* < 0.001). Once again it is in line with what has just been observed in other studies [51,52,53,55]. To our knowledge, no conflicting data has been published to date. Treadmill testing often elicits a more pronounced cardiovascular response due to the weight-bearing nature of the exercise, which may lead to a higher peak O_2_ pulse compared to cycle ergometry [29]. This difference can be attributed to the greater muscle mass engaged during running or walking, which can enhance stroke volume, and subsequently increase O_2_ pulse. Conversely, cycle ergometry may result in a more stable but lower O_2_ pulse, as the seated position and less muscle engagement may limit the cardiovascular response.

### 4.7. Oxygen Pulse Kynetics

In our study we observed a different pattern of the O_2_ pulse curve on the treadmill than on the cycle ergometer, a finding that, in our opinion, deserves further study in the future. In a healthy, moderately active individual, the O_2_ pulse is expected to exhibit a progressively increasing trend, typically culminating a plateau toward the end of the exercise.

Research indicates that many Fontan patients experience an early plateau in O_2_ pulse during exercise, regardless of the ergometer used [79]. Unexpectedly, in our study, this trend was observed on the treadmill to a significantly greater extent than on the cycle ergometer in the same subject.

In our opinion, this difference could be at least partly explained by the “pumping” action associated with the cyclic movement of the lower limbs during cycle ergometer tests, which probably promotes greater venous return, the main limiting factor for exercise in Fontan subjects, and thus a greater ability to increase systolic volume.

In addition, different peripheral O_2_ extraction kinetics might play a role, which in turn are related to the type of muscles involved in exertion, their oxidative capacity, and the greater or lesser habit of the population under study to its use. Supporting this hypothesis are a number of studies documenting how targeted exercise interventions have been shown to improve O_2_ pulse, suggesting that increased physical conditioning may lead to better cardiovascular responses during exertion [29]. This continued improvement in O_2_ pulse may indicate an adaptive response to training, allowing more efficient O_2_ utilization and potentially better stroke volume during exercise [79].

To the best of our knowledge, there are no other studies that describe the different kinetics of the O_2_ pulse as a function of the type of exercise in Fontan subjects. In light of the significant prognostic role of the O_2_ pulse, we believe that this aspect deserves to be investigated with further and targeted studies.

## 5. Limits

This study has, in our view, some limitations. Although the number of subjects enrolled is meaningful given the kind of pathology under investigation, an analysis on a larger and more age-homogeneous sample would be valuable. Furthermore, better standardization of the interval between the two tests would be desirable (with a shorter and more uniform time gap between assessments). In this study, however, such an objective could not be achieved for organizational reasons, as many patients lived far from the center and reported difficulties in attending closely spaced follow-up visits, in addition to the clinical need. Lastly, we tried to avoid rescheduling examinations already planned for other patients.

## 6. Conclusions

Many CPET parameters may differ depending on the type of ergometer used to perform the test. Several of these differences have already been documented in the literature, both in healthy individuals and in patients with HR or rTOF and appear to be confirmed in Fontan patients as well.

Although both ergometers have proven to be safe and reliable tools for assessing cardiopulmonary function, we believe these differences are particularly relevant in Fontan patients—not only for more accurate prognostic assessment but, even more importantly, for the personalized prescription of physical exercise. In this context, it is essential to re-evaluate the patient using the same type of ergometer in order to avoid under- or overestimation of changes in cardiopulmonary parameters

Moreover, our study highlights a novel finding, to our knowledge: the differing kinetics of the O_2_ pulse depends on the type of ergometer and protocol used—an observation that warrants further investigation.

## Figures and Tables

**Table 1:** Main anthropometric characteristics of 47 cases at first and second test.

	First Test					Second Test				
	Min	Max	25th Centile	50th Centile	75th Centile	Min	Max	25th Centile	50th Centile	75th Centile
Age (yrs)	10.3	37.2	14.0	16.4	19.5	11.2	38.0	14.0	16.5	21.4
Weight (kg)	38.0	96.0	51.3	58.0	65.0	39.5	97.0	50.1	56.6	66.8
Height (cm)	150.0	185.0	159.3	165.8	171.8	152.0	187.0	160.0	167.0	172.0
BMI	12.3	30.5	18.7	21.2	23.0	13.2	31.1	18.1	21.8	23.1
IPAQ	360	2500	550	950	1715	330	2515	578	1071	1770

BMI: body mass index; yrs: years.

**Table 2:** Main CPET parameters on treadmill and cycle ergometer.

	Cycle Ergometer					Treadmill				
	Min	Max	25th Centile	50th Centile	75th Centile	Min	Max	25th Centile	50th Centile	75th Centile
Peak HR (bpm)	126.0	191.0	153.0	167.0	177.0	137.0	196.0	157.0	169.0	180.0
Peak HR (% TMHR)	66.4	92.4	75.0	82.4	86.3	68.3	96.3	79.7	83.3	88.7
Resting SpO_2_ (%)	81.0	98.0	95.5	96.0	97.5	81.0	99.0	95.0	96.0	97.0
Minimum SpO_2_ (%)	79.0	98.0	89.5	92.0	95.0	76.0	98.0	90.0	92.0	95.0
Peak SpO_2_ (%)	79.0	98.0	89.5	92.0	95.5	77.0	98.0	90.3	92.0	95.0
Peak RER *	1.06	1.21	1.08	1.11	1.14	1.00	1.21	1.02	1.04	1.07
Peak VO_2_ (mL/min) *	842.0	1985.0	1132.0	1333.0	1498.5	935.0	2205.0	1217.0	1561.0	1743.8
Peak VO_2_ (mL/kg/min) *	13.0	33.8	19.9	23.2	25.2	17.8	39.3	23.2	25.6	29.4
Peak VO_2_ (% predicted) *	29.9	87.6	48.6	55.0	64.8	34.2	91.3	56.0	60.5	68.9
VO_2_ at VAT (mL/min) *	589.0	1340.0	788.0	902.0	1072.5	679.0	1862.0	1002.0	1260.5	1405.0
VO_2_ at VAT (mL/kg/min) *	10.0	23.5	14.1	15.9	18.6	12.9	29.1	18.4	20.7	24.2
VO_2_ at VAT * (% pred VO_2_ max)	32.0	61.1	34.0	38.7	44.5	28.3	76.2	44.7	49.7	56.9
Peak O_2_ pulse (mL/b) *	5.1	12.9	7.3	7.7	9.0	5.5	13.5	7.5	9.0	10.6
Peak O_2_ pulse * (% of predicted)	40.3	97.1	58.2	66.2	73.5	43.0	100.1	62.2	70.3	76.7
Slope VE/VCO_2_	26.9	58.7	32.5	36.0	41.9	22.9	53.7	33.6	37.2	43.9

HR: Heart Rate; bpm: Beat per Minute; TMHR: Theoretical Maximum Heart Rate; VATs: Ventilatory Anaerobic Thresholds, SpO2: Peripheral Arterial Oxygen Saturation, RER: Respiratory Exchange Ratio, VO_2_ max: Maximal Oxygen Consumption; VE/VCO_2_: Minute Ventilation/Carbon Dioxide Production, *: Statistical Significance with *p*-Values < 0.05.

## Data Availability

The raw data supporting the conclusions of the article will be made available by the authors on request.
